# Shining light on a deep-sea bacterial symbiont population structure with CRISPR

**DOI:** 10.1099/mgen.0.000625

**Published:** 2021-08-27

**Authors:** Maëva Perez, Bernard Angers, C. Robert Young, S. Kim Juniper

**Affiliations:** ^1^​ Université de Montréal, Québec, Canada; ^2^​ National Oceanography Center, Southampton, UK; ^3^​ University of Victoria, British Columbia, Canada

**Keywords:** CRISPR-based typing, conservation, marine microbiology, population genetics, symbiosis

## Abstract

Many foundation species in chemosynthesis-based ecosystems rely on environmentally acquired symbiotic bacteria for their survival. Hence, understanding the biogeographic distributions of these symbionts at regional scales is key to understanding patterns of connectivity and predicting resilience of their host populations (and thus whole communities). However, such assessments are challenging because they necessitate measuring bacterial genetic diversity at fine resolutions. For this purpose, the recently discovered clustered regularly interspaced short palindromic repeats (CRISPR) constitutes a promising new genetic marker. These DNA sequences harboured by about half of bacteria hold their viral immune memory, and as such, might allow discrimination of different lineages or strains of otherwise indistinguishable bacteria. In this study, we assessed the potential of CRISPR as a hypervariable phylogenetic marker in the context of a population genetic study of an uncultured bacterial species. We used high-throughput CRISPR-based typing along with multi-locus sequence analysis (MLSA) to characterize the regional population structure of the obligate but environmentally acquired symbiont species *Candidatus* Endoriftia persephone on the Juan de Fuca Ridge. Mixed symbiont populations of *Ca*. Endoriftia persephone were sampled across individual *Ridgeia piscesae* hosts from contrasting habitats in order to determine if environmental conditions rather than barriers to connectivity are more important drivers of symbiont diversity. We showed that CRISPR revealed a much higher symbiont genetic diversity than the other housekeeping genes. Several lines of evidence imply this diversity is indicative of environmental strains. Finally, we found with both CRISPR and gene markers that local symbiont populations are strongly differentiated across sites known to be isolated by deep-sea circulation patterns. This research showed the high power of CRISPR to resolve the genetic structure of uncultured bacterial populations and represents a step towards making keystone microbial species an integral part of conservation policies for upcoming mining operations on the seafloor.

## Data Summary

All supporting data have been provided within the article or through supplementary data files. Raw sequences used in this paper were deposited on GenBank under the BioProject PRJNA641184. Python scripts, R scripts, and the implementation of Kupczok and Bollback [1] method are publicly available at http://github.com/maepz/CRISPR_distance.

**Impact Statement**

CRISPR-based genotyping of bacterial strains is increasingly being used to track isolates of many human pathogens but can the CRISPR array be used for assessing the structure of natural populations of uncultured bacteria? In this study, we addressed this question by examining the local population structure of *Ca.* E. persephone, a keystone bacterial species, which enables high-biomass faunal communities to develop around deep-sea hydrothermal vents in the eastern Pacific Ocean. Sequences of the CRISPR array and other gene markers were obtained by high-throughput amplicon sequencing of heterogeneous assemblages of host-associated symbiont populations. The CRISPR marker revealed an unprecedented strain-level diversity for this species and its architecture accurately retraced the known symbiont phylogeny. At the local scale, the CRISPR-based population structure corroborated that of other hypervariable genes and indicated that limitations to dispersal contribute to the genetic partitioning of the free-living symbiont pools. Hence, we underscore the importance of fine-scale structure assessments for marine microbial populations and for this purpose, provide an implementation of Kupczok and Bollback’s probabilistic algorithm to estimate genetic distances between unique CRISPR arrays [1].

## Introduction

Marine bacteria and archaea perform vital marine ecosystem functions including primary productivity at the sunlit surface, remineralization and storage of carbon in the water column and the ocean’s interior through the biological carbon pump. They are also primary producers within the ocean’s dark interior, inhabiting environments such as hydrothermal vents and hydrocarbon seeps, where they utilize geochemical energy rather than sunlight to fix carbon. Given their fundamental roles in marine ecosystems, understanding the processes that govern microbial biogeographic distributions, community assembly and ecosystem function is a primary pursuit of marine microbial ecology. To achieve this goal, we need to understand how microbial distributions are determined by the interaction of physical and biological factors. Indeed, the paradigm formulated by Baas–Becking [2] that ‘everything is everywhere, but the environment selects’ is increasingly being challenged in marine systems [3]. Together with the collection of environmental data, the use of multiple hypervariable gene markers has provided a growing body of evidence suggesting that dispersal of microbes in the oceans is limited [4, 5], and that geographical isolation even affects bacteria at the local scale [6–8].

Assessments of the structure of bacterial populations are limited by the resolution of genetic markers. Gene markers with high conservation and low diversity lack the resolution to reveal fine-scale population structure. Previous studies have shown the very conserved 16S rRNA gene typically used to assess bacterial diversity is not variable enough to detect genetic diversity at the population level [6]. The clustered regularly interspaced short palindromic repeat (CRISPR), on the other hand, might provide the high-definition needed. The CRISPR locus is the adaptive immune system of prokaryotes [9]. It is composed of the Cas operon and the CRISPR array. The Cas operon contains genes responsible for editing the CRISPR array as well as genes with anti-viral functions [10]. The CRISPR array consists of short sequences (CRISPR spacers,~40 bp) that are complements to sequences in phage nucleic acids. These spacers are separated by short sequences of palindromic repeats that are species specific. The spacers constitute a historical record of the viral encounters of a particular lineage, because they always accumulate at the 5′ end of the CRISPR array. Furthermore, because each protospacer is randomly sampled from the virus genome, independent infections by the same virus phylotype would practically never result in the insertion of the exact same spacer sequence in two bacterial lineages [11, 12]. Various CRISPR loci are already in use for tracking the micro-evolution of pathogenic bacteria [1, 13–18] but the temporal dynamics of spacer acquisition and deletion are dependent on the viral context [19, 20] and are highly variable between species, casting doubt on the suitability of CRISPR as a universal hypervariable marker. For instance, Beauruelle *et al*. [18] discovered that new spacers were acquired by Group B *Streptococcus* in the span of a few years, whilst Savitskaya *et al*. [21] observed nearly identical CRISPR arrays in a present-day strain of *Escherichia coli* compared with one recovered from the guts of a 42 000 year-old frozen woolly mammoth. Yet, if CRISPR can be used for fine-scale strain typing, characterizing the local structure of bacterial populations may become possible even for uncultivated bacterial species and mixed populations.

Fine-scale strain typing would be particularly useful in conservation applications requiring knowledge of the structure and connectivity of populations of symbiotic bacteria. For example, in deep-sea chemosynthetic ecosystems such as hydrothermal vents, estimating symbiont population connectivity could inform the development of conservation strategies to mitigate the impacts of future deep-sea mining of polymetallic sulfide deposits. Estimations of macrofaunal connectivity are already an integral part of several frameworks aimed at assessing the resilience of proposed mining sites and developing preservation areas [22–24]. Assessments of microbial population structure and connectivity would be prudent in areas populated by keystone taxa that rely on obligate symbionts for their survival.

One such environment exists in the eastern Pacific Ocean, where hydrothermal vent communities are dominated by various species of gutless siboglinid polychaetes whose dense aggregations create niches for other faunal species. These tubeworms all rely on a single species of uncultured chemolithoautotrophic Gammaproteobacteria coined *Candidatus* Endoriftia persephone [25] for their nutrition. These bacterial symbionts are acquired *de novo* from the surrounding environment at each generation [26] by young tubeworm larvae during a short infection-phase and proliferate within the cells of a special hosting organ known as the trophosome. Despite the essential nature of the symbionts for these habitat-forming worms and therefore entire vent communities, little is known about the organization of their populations and their connectivity, particularly at the regional scale which is the relevant scale for conservation purposes.

Genetic studies investigating the phylogeography of *Ca*. E. persephone showed the symbionts associated with the species of the East Pacific Rise (*Riftia pachyptila* and *Tevnia jerichonana*, *Oasisia alvinae*) at tropical latitudes, and those associated with worms of the Juan de Fuca Ridge (*R. piscesae*, *Lamellibrachia* sp.) in the northeast Pacific, belong to two vicariant populations [27–29]. At different scales, previous studies of intra-host symbiont diversity in tubeworms inhabiting hydrothermal vents or hydrocarbon seeps [30–37], have consistently found low genetic diversity at the species-level but evidence for multiple strains. Such results also highlight the fact that conventional genetic markers do not provide a high enough resolution to uncover the true strain-level diversity of the symbionts. Also, it has been proposed the symbionts can escape the tissues of dead hosts and return to a free-living stage [38], potentially linking host-associated and free-living symbiont pools by strong gene flow. Furthermore, metagenomic sampling of environmental biofilms [36] and fluorescently-labelled *in situ* hybridization of colonization blocks (deployed for 1 year) [26] revealed that free-living *Ca*. E. persephone are most abundant in close proximity to host aggregations and almost undetectable away from zones of hydrothermal activity. Taken together, these observations suggest the genetic diversity of the symbionts is spatially structured and can be uncovered from host-associated populations with a suitable hypervariable genetic marker.

The goal of this study was therefore to evaluate the CRISPR sequence as an appropriate genetic marker for distinguishing multiple environmental strains of the uncultured vestimentiferan symbiont *Ca*. E. persephone. To address this objective, we used CRISPR along with four other gene markers to characterize the structure of *Ca*. E. persephone populations along the Juan de Fuca Ridge, where the symbiont species is associated with the host tubeworm species *Ridgeia piscesae*. Doing so, we assessed if the physicochemical conditions of the worm habitat contributed to symbiont population structure, and the extent to which the populations along the Juan de Fuca Ridge were connected.

## Methods

### Sampling design

**Fig. 1.**
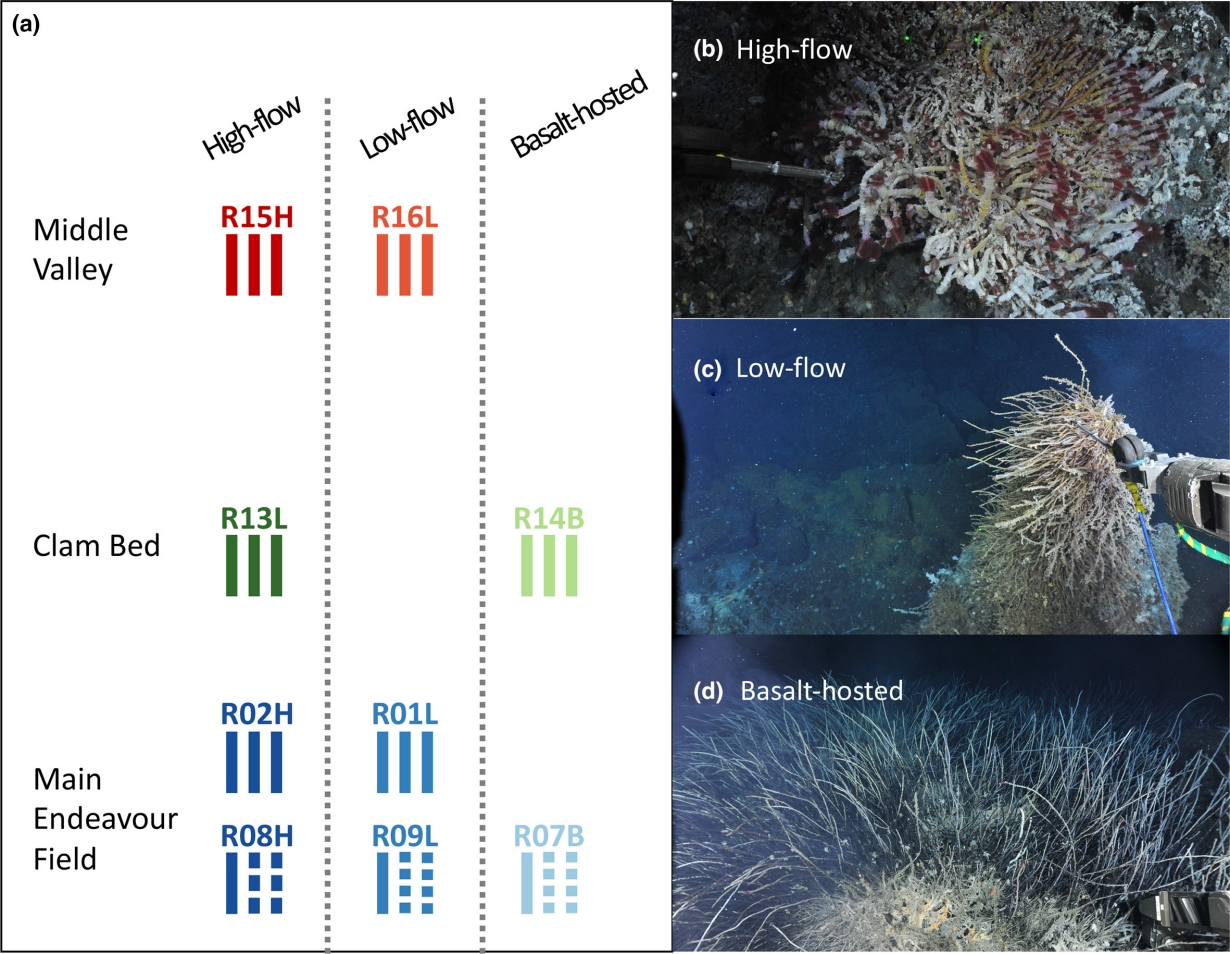
Environmental sampling design. (a) Schematic representation of the sampling design. Each bar represents an individual worm from which a symbiont population was sampled. Segmented bars represent worms that were sectioned. Sampling sites within individual vent fields were separated by ~10 m. Clam Bed is located ~2 km north of the Main Endeavour Field and Middle Valley is ~60 km further north. (b) Tubeworm aggregation at site R08H, a typical a ‘high-flow’ habitat. (c) Tubeworm aggregation at the ‘low-flow’ site R09L. (d) Tubeworms of a ‘basalt-hosted’ habitat (site R07B).

Populations of environmentally acquired *Ca*. E. persephone were sampled from their *R. piscesae* hosts in three active hydrothermal venting regions separated by increasing N-S distances along the Juan de Fuca Ridge: Main Endeavour Field (MEF), Clam-Bed (CB) and Middle Valley (MV). Within each region we sampled contrasting habitats, which were identified from the morphotypic appearance of the individual *R. piscesae* hosts, which exhibit environmentally driven phenotypic plasticity [39] (Fig. 1, Table S1, available in the online version of this article). The first habitat called ‘high-flow’ is typically located on sulfide edifices, close to points of vigorous discharge of hydrothermal fluids (Fig. 1b). The average fluid temperature in the tubeworm aggregations growing in this environment was 10 °C at the level of the gills and 37 °C at the base of the tubes. The second environment (‘low-flow’) is also located on sulfide chimneys but away from discharge zones (Fig. 1c). Temperatures in the ‘low-flow’ tubeworm bushes ranged from 4 °C (gill level) to 16 °C (base). Finally, we referred to the third habitat type as ‘basalt-hosted’. This environment was located in the vicinity of the hydrothermal edifices, where the venting fluids emerged from basalts rather than sulfide accretions (Fig. 1d). Temperature recorded at both the plume and base level of the tubeworm bushes in these peripheral habitats was around 2 °C, slightly above the ambient seawater temperature of 1.8 ˚C. Temperature has been shown to be a reliable proxy for sulfide concentrations in Juan de Fuca Ridge hydrothermal vent fluids [40].

Using the intracellular symbionts to assess the diversity of the *Ca*. E persephone population as a whole is problematic because we do not know if the host-associated symbionts are representative of the free-living pool. For example, the infection process likely represents a bottleneck event and it is not known if discriminatory selection occurs during the development of the trophosome. To overcome this limitation, we sampled multiple worms from each aggregation/sampling site, and intra-host variation was assessed by partitioning the trophosomes of several worms from the MEF sites into three to four transverse sections (Fig. 1a). Because of budget constraints, only three replicate symbiont populations (i.e. three individual host worms) were examined per site. For the same reason, intra-host variation could not be assessed for all worms so we restricted this analysis to a few host worms from MEF in order to compare intra-host variation across all three habitats (Fig. 1).

### Sample collection and DNA extraction

Tubeworm assemblages typical of the three contrasting environmental conditions, were sampled in June 2013 and June 2016 during two deep-sea expeditions that deployed remotely operated vehicles (ROVs) from research vessels. The first, on board the R/V Thomas G. Thompson, used the ROV Oceaneering Millennium, whilst the second, on the CCGS John P. Tully, used the ROV ROPOS (Table S1). Worms collected in 2013 were processed upon recovery to the surface vessel, whereas those collected in 2016 were individually packed, frozen at −80 °C and later dissected in the laboratory. The individual worms were carefully removed from their tubes and treated with lysozyme and DNAse according to Elsaied and Nagamura [41] to remove epibiotic contamination. Subsequently, the trunks of the worms were separated, and for some individuals split into three to four segments (see sampling design), before being placed in 95 % ETOH pending DNA extraction. Trunk sections were later finely chopped with scissors and homogenized by strong vortexing to release symbionts cells from the trophosome tissues. We then collected and precipitated a subsample of each of the symbiont-enriched suspensions. DNA was extracted using the phenol-chloroform method followed by ethanol precipitation [42].

### Genetic sampling and sequencing

To confirm that *Ca*. E. persephone was the only symbiont species within *R*. piscesae, we amplified and sequenced the hypervariable region V4 of the bacterial 16 s rRNA gene using universal primers. To assess the intra-specific genetic diversity of the symbionts we amplified and sequenced a complete CRISPR array previously found on the scaffold KQ557120 (48 218 .48978) (start-end positions) in the assembly ‘*Ridgeia* 1 symbionts’ (GenBank accession LDXT01). Additionally, we performed a multi-locus sequence analysis (MLSA) by sequencing three additional protein-coding genomic regions. Rather than using genes typically employed in MLSA analyses (e.g*. recA*, *gyrB, rpoB, rpoD, groEL, atpD* [43]), we selected the protein-coding sequences that had the highest potential for displaying polymorphism based on SNPs previously detected in the metagenomics sequences from the trophosomes of one, and a pool of five individual tubeworms, respectively [34]. Candidate genes had to be uniquely represented in the *Ca*. E. persephone genome, belong to a well-defined Cluster of Orthologous Genes (COG) category, have multiple SNPs within 600 bp of each other but no indels. Six genes fitted these criteria but only three were successfully amplified: *lpxA*, *pleD* and *tufB*.

Libraries were prepared according to Génome Québec guidelines. A first PCR was performed to amplify the genomic regions of interest. We used gene-specific primers that carried the CS1 and CS2 universal overhangs. These extra 22 bp sequences allowed for the attachment of sample-specific barcodes during a second PCR round. Primer sequences and PCR conditions are presented in Table S2. Ultimately, 35 libraries of pooled barcoded amplicons (one barcode per sample) for the polymorphic gene fragments (i.e. 16S rRNA V4 region, *lpxA*, *pleD* and *tufB*) were then sent to Génome Québec for sequencing on the Illumina MiSeq 2500 platform (1 % of a lane), and 41 libraries for the CRISPR PCR products were sequenced on one PacBio SMRT cell.

### *In silico* haplotype detection

#### Gene amplicons

Sequencing of the four pooled gene amplicons (16S rRNA gene- V4 region, *lpxA*, *pleD* and *tufB* gene fragments) yielded between 1385 and 3199 reads per sample. Of these, 70 % were concordantly mapped to the reference genome. In order to isolate the amplicons of different loci, the paired-end reads were mapped onto the reference genome using bowtie2 v2.3.2 [44] with the following parameters: -D 15 R 2 –N -L 20 –I S,1,0.75 –dovetail –qseq –X 600. For each gene, the mapped reads were then extracted with samtools v1.9 [45] and the bamtofastq program from bedtools v2.27.1 [46].

For the hypervariable region V4 of the bacterial 16 s rRNA gene, we extracted both the reads that mapped to the 16S rRNA gene and those that did not map to the reference at all. These sequences were then together processed with the software package Divisive Amplicon Denoising Algorithm 2 (DADA2 v1.17.0) [47] in R, according to the pipeline tutorial version 1.6 (https://benjjneb.github.io/dada2/tutorial.html). For the other housekeeping genes (*lpxA*, *pleD* and *tufB*), polymorphic positions across all mapped reads were initially detected with VarScan v2.3.9 using the following parameters: --min-coverage 100 --min-reads2 10 --min-avg-qual 25 --min-*var*-freq 0.01 [48]. All SNPs identified matched known SNPs from the reference (Table S2) and no additional variable sites were found. The putative ancestral haplotypes for these genes was determined from their respective nucleotide sequences in the genome of *Ca*. E. persephone associated with tubeworms of the East Pacific Rise [49]. Then, extracted reads were merged with bbmerge (BBmap v38.70) [50] using 3′ quality trimming, transformed to fasta format conservatively changing low quality (< 28) nucleotides to Ns, and aligned with Muscle v3.8.31 using default parameters [51]. Finally, the alignments were truncated to the two SNP positions and the haplotype frequencies were counted with a custom python script. With the exception of two samples for the *tufB* amplicon, which failed to amplify, the final minimum coverage on the gene markers reached 94X (average 286, 579, and 378X for *lpxA*, *pleD* and *tufB*, respectively)

#### CRISPR array

**Fig. 2.**
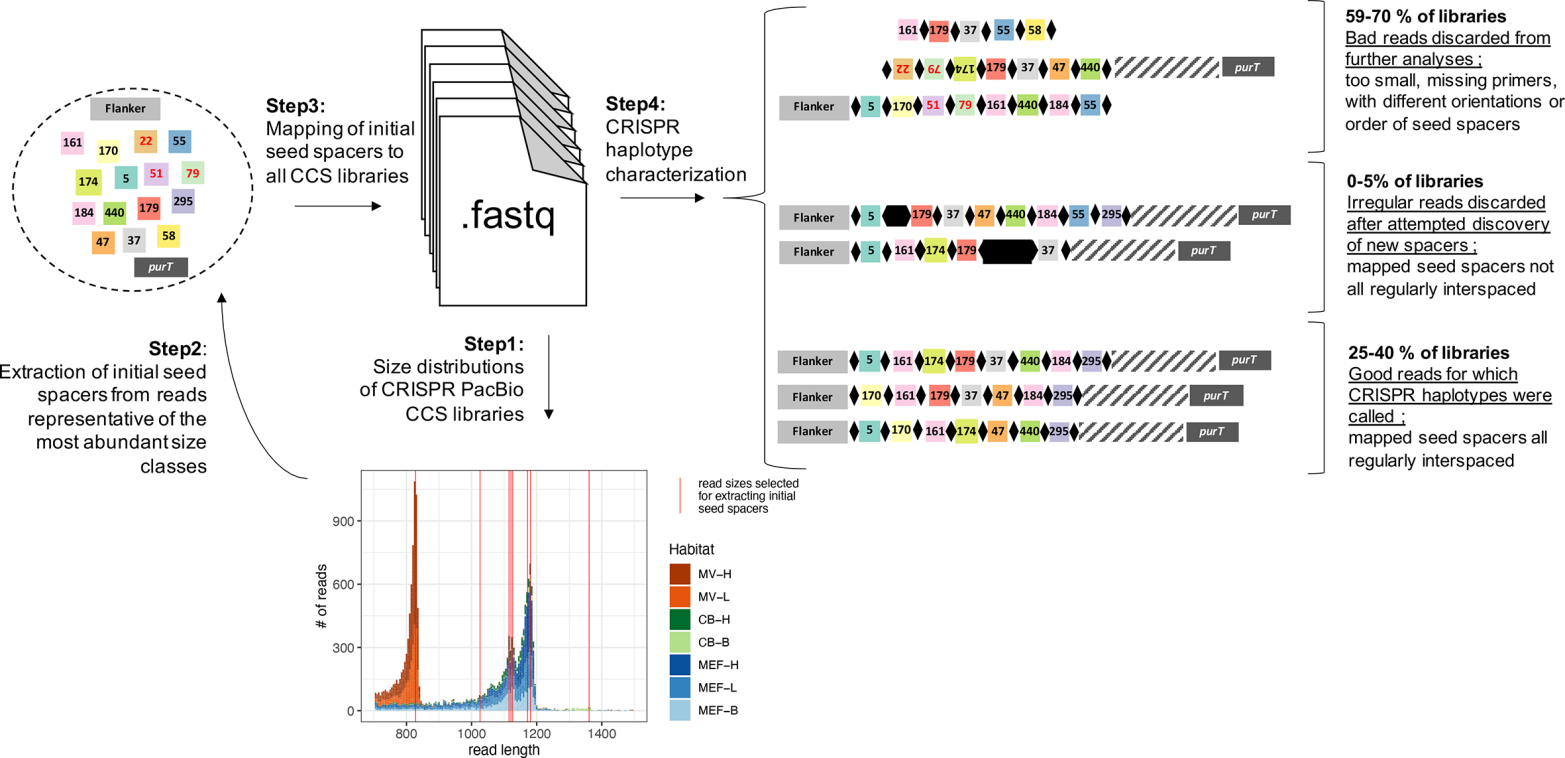
Schematic representation of the workflow for CRISPR haplotype detection.

A total of 35 840 high-quality PacBio reads from CRISPR amplicons were generated through circular consensus sequencing (CCS). Initial attempts at direct spacer detection in the CCS reads using two available methods designed for Illumina reads, Crass v0.3.12 [52] and MetaCRAST [53], yielded too many artifactual spacers because of the higher error rate of the CCS reads compared to Illumina. We thus adopted a different approach shown in Fig. 2.

First, we established read-length distributions for each sample. A small subset of reads representative of the most abundant size classes was then extracted and a set of seed spacers were identified with Crass v0.3.12 [52] using all default parameters except kmer length, which we picked to match the length of the repeat sequence (-K30). Of the resulting 15 seed spacers identified, 11 had been previously described for this array [34] and three were new. Next, the seed spacers along with subsequences of the two primers used for PCR amplification of the whole CRISPR array were mapped back to all CCS reads using an approximate string-matching algorithm implemented in the python package fuzzysearch 0.7.2. Near-matches with a minimum of 85 % identity (i.e*.* a maximum of 5 nucleotide mismatches) between the spacer sequence and the read were tolerated to account for sequencing errors and mutations. We discarded from further analysis dubious reads, which were missing at least one of the primer sequences, were less than 33 bp (the approximate size of a single spacer), or whose mapped seed spacers did not all have the same orientation or were properly ordered. Discarded reads represented 59–70 % (22 738 reads in total) of the CCS libraries and most (>90 %) were excluded because of missing primer sequences. To assess whether additional spacers (not detected from the initial read subset) were present in the samples, we flagged all reads for which mapped seed spacers were not regularly interspaced (0–5 % of the libraries; 439 reads in total), as putatively containing new spacers. The sequences of these putative new spacers including their bordering repeats were extracted to a new fastq file and processed with Crass v0.3.12 using the same parameters as for the initial seed spacer search [52]. Only three distinct spacers were found and all had matches in the initial seed spacer set but with a score slightly below the conservative threshold of 85 % identity we used for initial mapping. Because they represented only a small proportion of the libraries and had lower quality, these reads were also excluded from further analyses. The remaining 25–40 % of the libraries contained the highest quality reads for which mapped seed spacers were all regularly interspaced. It is in this read set (12 663 reads) that unique arrays of CRISPR spacers (also referred to as CRISPR haplotypes) were called.

### CRISPR phylogeny

We estimated the genetic distance between pairs of CRISPR haplotypes by implementing the probabilistic algorithm described by Kupczok and Bollback [1] for estimating the parameters underlying the ordered independent spacer loss model. This model assumes spacers are independently added at the leader end of the array and independently lost one at the time throughout the array. The parameters estimated are the insertion to deletion rate ratio and the divergence time between each haplotype pair and its most recent common ancestor. The resulting distance matrix between the haplotypes and their most recent common ancestors was used to reconstruct the phylogeny of the CRISPR arrays. To do so, we used a modified version of the rooted neighbour joining method presented in Kupczok and Bollback [1], which does not allow for negative branch lengths. As in Kuhner and Felsenstein [54], each negative branch was corrected to zero during tree construction and the corresponding difference was added to the adjacent branch length in order to preserve the total distance between adjacent pairs of terminal nodes. The genetic distances between haplotype pairs were then computed from the distances between pairs of terminal nodes in the tree.

### Population structure

Analyses of population structure were performed for the CRISPR array and each gene amplicon independently in R using the package ‘Proppr’ v2.8.6 [55]. Each read was considered as an individual (i.e. a unique symbiont cell; ignoring PCR amplification biases) and each individual worm host represented a discrete bacterial population.

Minimum-spanning trees based on the previously estimated genetic distances between CRISPR haplotypes were constructed using the function *poppr.msn*. The function first computes a minimum spanning tree from a graph representation of an adjacency matrix (here, that of the pairwise CRISPR distances) and then adds population parameters as attributes to this tree.

Hierarchical AMOVAs [56] were performed with the wrapper *poppr.amova,* which uses the *amova* function from the ‘ade4’ package [57]. The following levels were tested: regions, habitats, sampling sites, individual hosts, trophosome sections within hosts, technical replicates. A permutation test with 1000 permutations (function *ade4:randtest*) was used to assess the statistical significance of the various covariance components (i.e. the hierarchical levels). To assess the concordance of the symbiont population structures according to the CRISPR array and each of the other gene fragments, we performed Mantel tests on their respective matrices of pairwise population differentiation. For the gene amplicons, the *F*_ST_ index computed in Arlequin v3.5 [58] was used as a measure of distance between pairs of symbiont populations and non-significant *F*_ST_ values were treated as 0 (function *mantel.test* from the ‘ape’ R package) or removed from the correlation coefficient computation (function *mantel* from the ‘vegan’ R package).

## Results and Discussion

### Unlike DNA barcoding using 16S rRNA gene, CRISPR-typing uncovers the high diversity of environmental symbiont strains

**Fig. 3.**
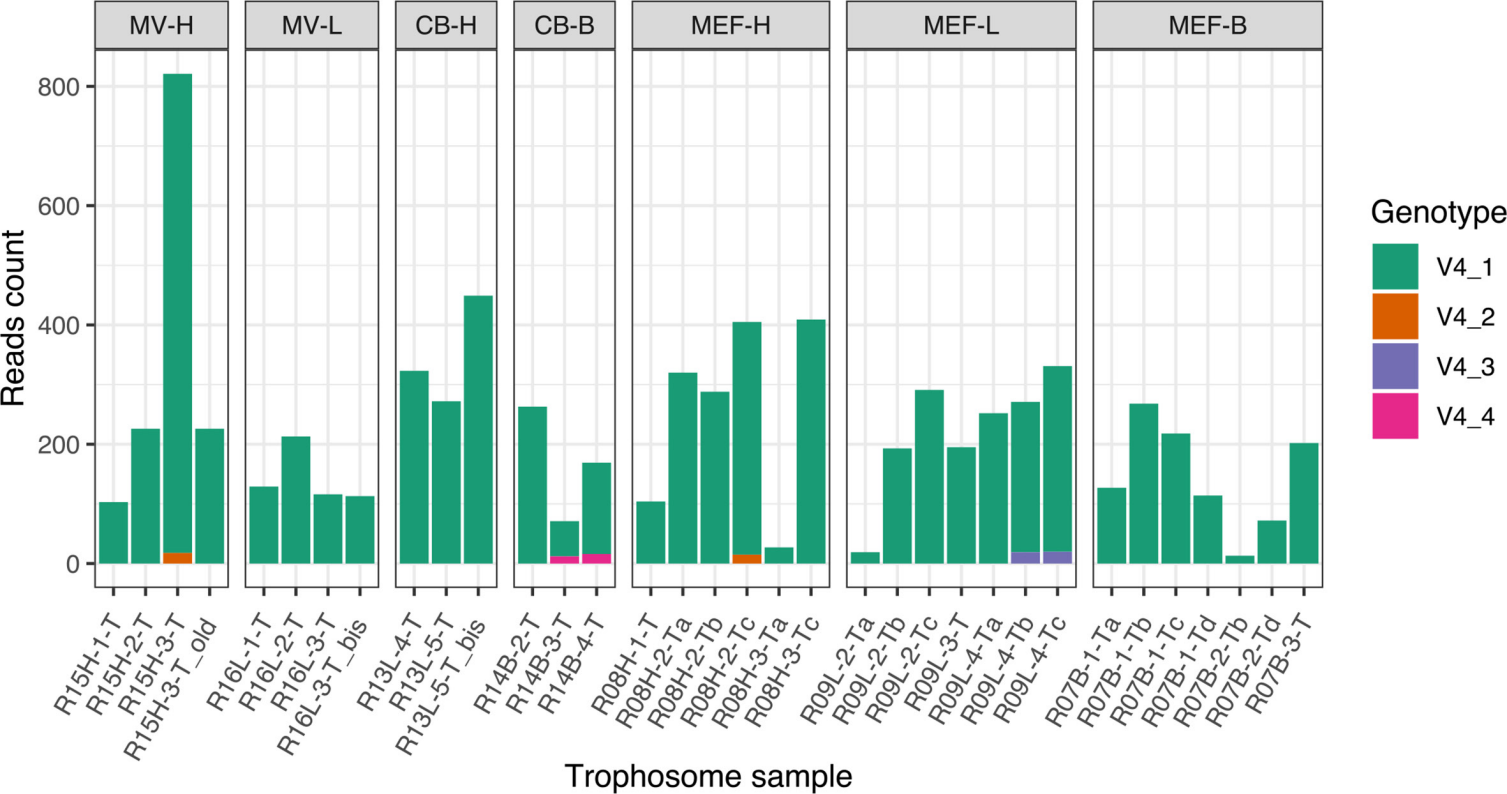
Symbiont genetic diversity according to the 16S rRNA gene16S rRNA genehypervariable V4 region. Samples suffixed ‘old’ and ‘bis’ are technical duplicates. Only high confidence genotypes, which accounted for ten reads in at least one of the samples are represented. The minimum nucleotide identity between genotypes was 81 % between V4_2 and V4_3. All genotypes were identified as *R. piscesae* symbionts by nucleotide blast against the NCBI’s nucleotide collection. MV: Middle Valley; CB: Clam-Bed; MEF: Main Endeavour Field; H: high-flow, L: low-flow, B: basalt-hosted.

The sequencing of the 16S rRNA gene- V4 amplicon yielded 8571 paired-end reads in total. The average per-sample coverage was 226X but there were important disparities across samples (Fig. 3). Using these reads together with all unmapped amplicons, we used dada2 to determine the symbiont genetic diversity.

We identified four bacterial phylotypes with a minimum nucleotide identity of 81 % between V4_1 and V4_4 (Fig. 3). The phylotypes V4_1 was identical to that of the reference symbiont genome [29] and overwhelmingly dominated the trophosome assemblages. A blast search of the other phylotypes against NCBI’s database identified them all as known endosymbionts of *R. piscesae*. Although great precaution was taken to reduce contamination, it is unclear given their low abundance and considerable divergence to the dominant endosymbiont phylotype if these alternative rare taxa are truly from host-associated bacteria. Regardless, these observations support the hypothesis that host-symbiont molecular interactions and microbial competition prevent the infection and intra-host proliferation of excessively divergent phylotypes [36].

**Fig. 4.**
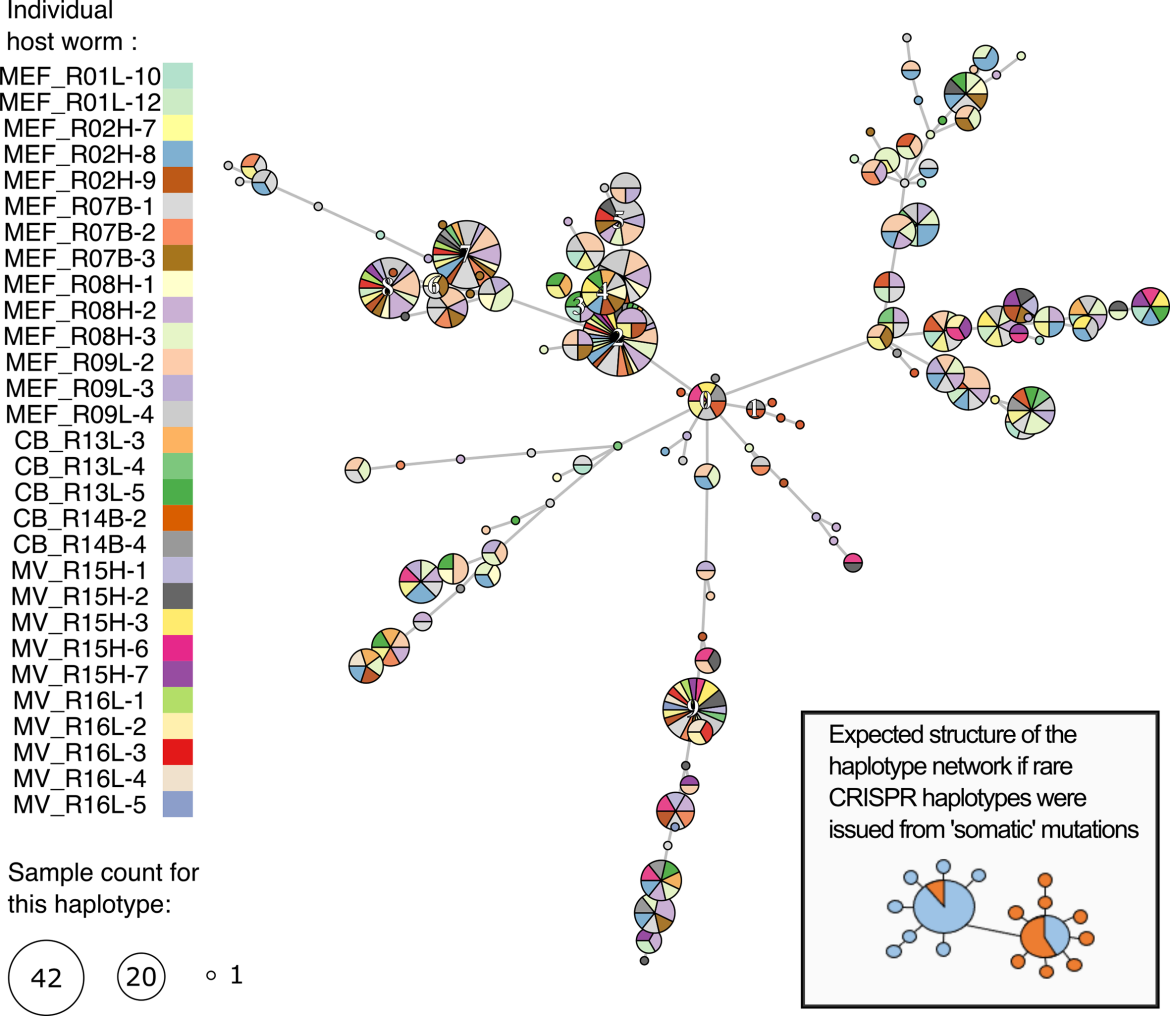
Minimum spanning tree for the CRISPR haplotypes coloured according to individual hosts. The sizes of the circles represent the number of trophosome samples within which a particular haplotype was found. The 10 most abundant haplotypes are labelled.

With 123 distinct CRISPR arrays detected, the hypervariable CRISPR region revealed a much higher symbiont genetic diversity than the 16S marker. Between 3 and 32 (median=11) distinct CRISPR haplotypes were found in each individual host. The majority of these haplotypes were in very low abundance; two thirds or more of the haplotypes were represented in fewer than 5 % of the reads. Nonetheless, rare haplotypes could not be identified as somatic variants (i.e. strains resulting from within-host mutations). Indeed, examination of a minimum spanning tree showed most CRISPR haplotypes were shared amongst host worms (Fig. 4). Furthermore, haplotypes present in a single host did not form phylogenetic clusters as would be expected from clonal populations (Fig. 4). Hence, we conclude that these haplotypes probably reflect the diversity of the environmental strains of *Ca*. E. persephone.

### The architecture of the CRISPR array retraces the known symbiont phylogeny

**Fig. 5.**
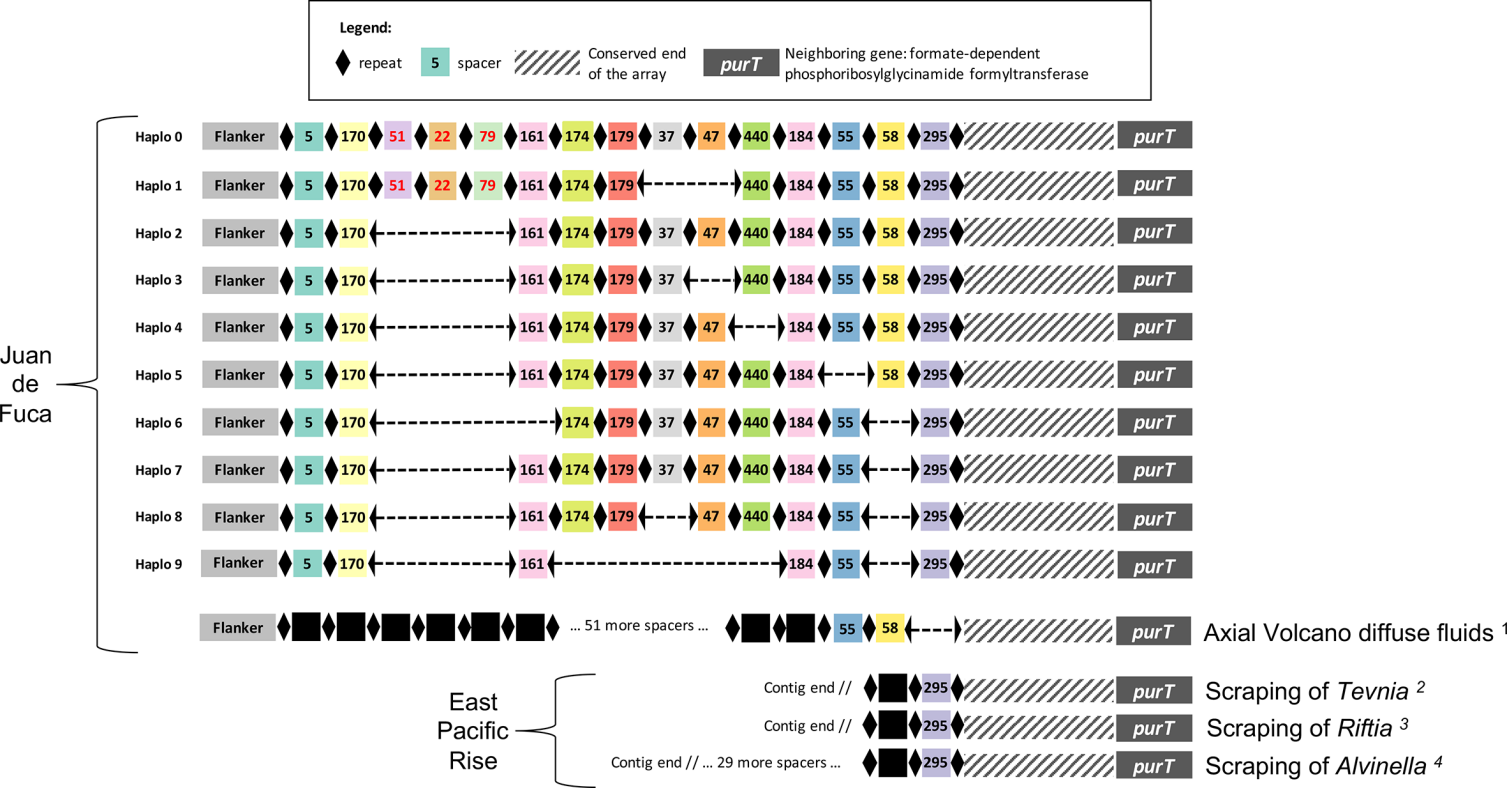
Schematic representation of the main CRISPR arrays of symbionts on the Juan de Fuca Ridge and East Pacific Rise. These arrays were present in at least 5 % of the CCS reads in at least one trophosome sample and represented more than 95 % of the reads overall. Repeats are represented by lozenges and each unique spacer is represented by a coloured square and ID number. Spacer ID numbers were randomly assigned and refer to those used in Perez and Juniper [34]. Spacers newly detected in this study are identified by red ID numbers. Dashed line between repeats represent missing spacers in the arrays. Black squares represent unique spacers in the metagenomic datasets. Note that while the most ancient spacer identified was spacer 295, a region of about 360 bp (hatched segment) extended from this spacer to the start of the following gene (*purT*). We suspect spacers in this region were not detected because accumulated mutations in the repeats rendered them unrecognizable by the spacer detection software. IMG scaffold references: 1 Ga0105700_1013652 ; 2 Ga0256846_1000282, Ga0256846_1005979, Ga0256846_1868271, Ga0256846_1117288; 3 Ga0256845_1170191; 4 Ga0256843_1000224.

Examining the structure of all the CRISPR arrays recovered from the Juan de Fuca Ridge tubeworms, we found that the genetic diversity amongst the symbiont haplotypes is defined by various spacer deletions from the longest array. The longest array thus represents the most ancestral state amongst the CRISPR arrays we sampled (Fig. 5).

Surprisingly, we observed no new spacers at the array’s leading end where insertions would be indicative of recent viral infections. Other known examples of deletion-driven CRISPR polymorphism are found in *E. coli, Salmonella* and *Klebsiella* species [59–61]. It is possible the immune function of CRISPR has been lost in this species. At evolutionary time scales, metabolic functions that are associated with a free-living lifestyle such as these providing immunity against phages are the first to be lost when symbionts are transitioning from being environmentally acquired facultative partners to becoming obligate and vertically transmitted organelle-like organisms [62–64]. Furthermore, in *R. pachyptila* the CRISPR-cas operon of *Ca*. E. persephone does not seem to be expressed within the trophosome [49, 65, 66]. Yet, in *Ca*. E. persephone the maintenance of the structural integrity of the arrays suggests the CRISPR/cas system may not be completely defunct and could hold instead an alternative function, notably during the free-living stage of the symbionts. Indeed, CRISPR/cas systems in free-living *Ca*. E. persephone may be involved in a number of physiological responses to environmental stress [67] and promote host colonization [68–70].

We then assessed if any of the 15 identified spacers had previously been sequenced by blasting them against JGI’s IMG-MER database. Three spacers found a match in contigs from other bacterial genomes and metagenomes. Because these contigs possessed the conserved end of the CRISPR array up to the *purT* gene, we are confident they are DNA fragments of *Ca*. E. persephone. The oldest identified spacer (spacer 295) in our dataset was also present in contigs from three metagenomes of bacterial communities sampled from the surface of three species of polychaete worms from the East Pacific Rise: *T. jerichonana, R. pachyptila* and *Alvinella pompejana*. Hence, spacer 295, the most ancient spacer in the array was most likely acquired before the vicariance of the Juan de Fuca Ridge and East Pacific Rise symbiont populations, following separation of the two ridges by the fragmentation of the Farallon plate about 30 million years ago [29, 71].

Given the presence of spacer 295 in many samples from the East Pacific Rise, it is surprising this spacer was not found in the reference genomes of *Tevnia*- and *Riftia*-associated symbionts [49]. We suspect this is due to the fragmented and incomplete nature of these assemblies; the CRISPR locus was consistently found at contig ends.

The next two most ancient spacers (spacers 58 and 55) were both detected in a contig from a metagenome of diffuse hydrothermal fluids at Axial Seamount, a shallower site on the Juan de Fuca Ridge located about 200 km south of the Main Endeavour field. None of the other spacers inserted at the leader end of the arrays were shared between Axial Seamount and the northern Juan de Fuca Ridge populations, suggesting a lack of connectivity between these populations which is supported by genetic analyses of their *R. piscesae* host [72, 73]. Together, these results show the CRISPR array retraces the known phylogeny of the symbiont over millions of years.

### The local symbiont population structure according to CRISPR is corroborated by other hypervariable gene markers

Three additional housekeeping gene fragments were amplified from the same individual hosts examined for CRISPR; *lpxA*, *pleD* and *tufB* (see Supplementary Material). These genes were specifically chosen because they exhibited polymorphism in metagenomic data [34] but only two of the three were informative. The genetic diversity of *tufB* was characterized by two pairs of haplotypes in similar proportions, which we suspect results from sequencing two recombining paralogous sequences [74–76]; the second copy may have been missing from our incomplete reference assembly causing us to mistakenly consider this gene as single copy gene. Haplotype frequencies for the two informative gene markers (*lpxA* and *pleD*) and the CRISPR array were used to compute matrices of population differentiation based on pairwise F-statistics. Mantel tests (see Supplementary Material) confirmed the haplotypes across *lpxA* and *pleD* markers exhibit a significant degree of covariation and revealed strong concordance to the symbiont population structure uncovered with CRISPR.

### Barriers to connectivity rather than local environmental conditions seem to be responsible for partitioning the symbiont populations

CRISPR-based inferences are presented here and corroborating AMOVA results for the other gene amplicons are provided in the Supplementary Material. Within the Main Endeavour Field (MEF) region, two independent tubeworm aggregations for each of the high- and low-flow environmental conditions were sampled to discriminate between habitat and aggregation-specific variation (Table 1). Furthermore, the symbiont housing organs of several individuals were partitioned into three to four sections to assess intra-host variation.

**Table 1.** Phylogenetically informed hierarchical AMOVA for symbiont populations in the MEF. The Lingoes transformation was applied to the haplotype distance matrix to satisfy the Euclidian criterion

Hierarchical level of variation	Df	Sum Sq	Mean Sq	Sigma	%	*P* value	*F*-statistic
Between habitat	2	18 148	9074	3.70	11.44	N.S.	*F*_Flow-Total_: 0.11
Between sites within habitat	2	2173	1086	−2.90	−8.96	N.S.	*F*_Site-Flow_: −0.10
Between hosts within sites	9	33 714	3746	7.80	24.11	0.009	*F*_Ind-Site_: 0.25
Between sections within hosts	12	9596	800	2.08	6.42	N.S.	*F*_Section-Ind_: 0.09
Between duplicates within samples	1	154	154	1.08	3.33	0.001	*F*_Samples-Section_: 0.05
Within samples	6159	126 799	21	20.59	63.66	0.001	*F*_Samples-Total_: 0.36
Total	6185	190 584	31	32.34	100.00		

### Within and between host variation

Significant variation in the composition of the symbiont strain assemblages was found across the length of the trophosome with the gene markers but not with CRISPR, the marker displaying a greater allelic diversity. This indicates that small contrasts in the symbiont composition along the length of the trophosome are likely exacerbated when genetic resolution is low and probably result from the random distribution of the different symbiont strains in the trophosome. In other words, even though the different symbiont strains may not be homogeneously distributed within the host housing organ, they are not partitioned in a specific way along its antero-posterior axis.

AMOVA analyses within MEF revealed that individual worms from the same aggregation could host markedly different strains; between-hosts variance accounted for nearly 25 % of the total variation (Table 1). This differentiation supports the hypothesis that the infection is not a continuous process but occurs during a small window of time [77].

### Between habitat variation

At Clam-Bed (CB), the allelic composition of symbionts from the high-flow and basalt-hosted worm populations was markedly different (see Supplementary Material) and could be the result of larger differences in age between the two tubeworm populations. The basalt-hosted worms at this site are known to be at least several decades old [78], and closely related species living in similar environmental conditions may live for centuries [79]. In contrast, the high-flow worms have likely colonized the CB chimney much more recently [80, 81]. Supporting this hypothesis, we found the ancestral haplotypes for all three symbiont genes (CRISPR, lpxA and pleD) were predominant amongst CB’s basalt-hosted populations (see Supplementary Material) suggesting the symbionts were established in the basalt-hosted tubeworms before hosts from the high-flow environment acquired theirs. It is also possible that the ancestral haplotypes of *Ca*. E. persephone were uniquely sustained in high abundance amongst the free-living population at this site. However, our fine-scale genetic survey revealed that while the symbiont populations were structured at the scale of a vent field, this structure was not driven by differences between habitats.

Indeed, broad environmental conditions associated with the concentration of hydrothermal discharge in the worms’ habitat generally did not significantly explain the variation observed in the data even when controlling for regional variation or excluding the highly homogeneous Middle Valley sites (Tables 1 and 2).

**Table 2.** Phylogenetically informed hierarchical AMOVA for symbiont populations in the MEF, CB and MV. The Lingoes transformation was applied to the haplotype distance matrix to satisfy the Euclidian criterion

(a) All regions							
	Df	Sum Sq	Mean Sq	Sigma	%	*P*-value	*F*-statistic
Between regions	2	446 357	223 178	59.39	66.87	0.001	*F*_Region-Total_ : 0.67
Between habitats within regions	4	31 696	7924	1.41	1.58	N.S.	*F*_Flow-Region_ : 0.05
Between hosts within habitats	23	91 384	3973	10.17	11.45	0.001	*F*_Ind-Flow_ : 0.36
Within hosts	12 633	225 439	18	17.85	20.09	0.001	*F*_Ind-Total_ : 0.80
Total	12 662	794 875	63	88.81	100.00		
Between habitats	2	63 857	31 928	0.76	1.17	N.S.	*F*_Flow-Total_ : 0.01
Between hosts within habitats	27	505 579	18 725	46.81	71.55	0.001	*F*_Ind-Flow_ : 0.72
Within hosts	12 633	225 439	18	17.85	27.28	0.001	*F*_Samples-Total_ : 0.73
Total	12 662	794 875	63	65.42	100.00		
			**(b) Without MV**				
Between regions	1	9309	9309	2.25	5.33	N.S.	*F*_Region-Total_ : 0.05
Between habitats within regions	3	25 887	8629	0.34	0.80	N.S.	*F*_Flow-Region_ : 0.01
Between hosts within habitats	15	75 356	5024	15.52	36.74	0.001	*F*_Ind-Flow_ : 0.39
Within hosts	7284	175 774	24	24.13	57.13	0.001	*F*_Ind-Total_ : 0.43
Total	7303	286 325	39	42.24	100.00		
Between habitats	2	18 183	9091	−0.57	−1.41	N.S.	*F*_Flow-Total_ : −0.01
Between hosts within habitats	17	92 368	5433	16.82	41.65	0.001	*F*_Ind-Flow_ : 0.41
Within hosts	7284	175 774	24	24.13	59.75	0.001	*F*_Samples-Total_ : 0.40
Total	7303	286 325	39	40.38	100.00		

### Between region variation

Variance in the symbiont strain diversity appears to reflect general patterns of connectivity along the Juan de Fuca Ridge rather than environmental selection. Regional differences between Middle Valley and the two Endeavour sites (CB and MEF) accounted for most of the regional variance (67 %, Table 2a) whereas the symbiont meta-populations were not significantly differentiated between CB and MEF (Table 2b).

Our results interpreted alongside those of the host populations suggest that both host larvae and symbiont cell dispersal depend on patterns of deep-sea circulation that restrict connectivity across disjointed axial rift valleys but maintain it within them. Young *et al*. [72] and Puetz [73] found a similar structure for the host populations. In both studies, tubeworm populations from Middle Valley at the northern extremity of the Juan de Fuca Ridge, which is a topologically isolated basin [82], were distinct from those of the Endeavour Segment to the south. Furthermore, Puetz [73] showed high gene flow between host populations inhabiting high- and low-flow habitat types.

**Fig. 6.**
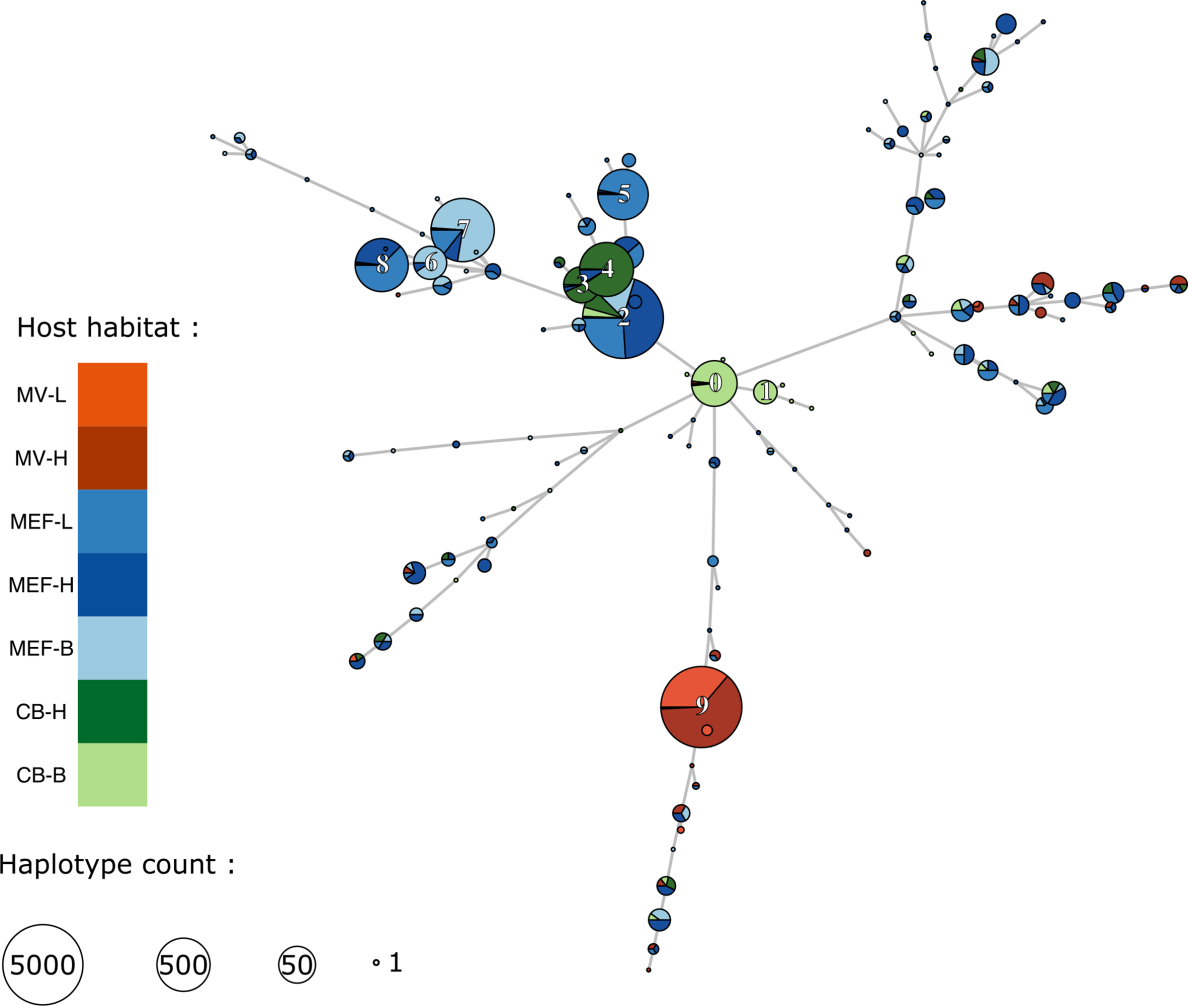
Minimum spanning tree for the CRISPR haplotypes coloured according to habitats. The sizes of the circles represent the number of sequenced reads. The 10 most abundant haplotypes are labelled.

It is noteworthy that in addition to their apparent isolation, symbiont populations from Middle Valley exhibited a surprisingly low diversity. All host individuals in this region were associated with a single symbiont strain identified by all three of the suitable hypervariable markers (CRISPR, *lpxA* and *pleD*) (Fig. 6 and Supplementary Material). This homogeneity likely reflects that of the environmental infection pool in this region. Little is known about the dependence of tubeworm recruitment on the resident environmental symbiont population or how important resident hosts are in maintaining this pool. If *R. piscesae* recruitment in Middle Valley is dependent on this symbiont strain or if robust host populations must be present to seed and maintain the symbiont populations, these worms and the associated communities that depend on them could be extremely vulnerable to disturbance from mining activities. Hence, our results highlight the fundamental importance of better understanding the diversity and connectivity of natural populations of obligate microbial symbionts. As the International Seabed Authority is drafting the first regulations for hydrothermal vent mining, we argue that it is imperative for such keystone bacterial species to be taken into account within conservation schemes.

## Conclusions

Characterizing and comparing natural heterogeneous bacterial populations at the strain level is challenging. We have demonstrated that the CRISPR array is a genetic marker fit-for-purpose for the uncultured chemoautrophic symbiont species *Ca*. E. persephone. In our study, the CRISPR array retraced the known symbiont phylogeny over millions of years but also allowed for discrimination amongst very closely related lineages. The CRISPR haplotypes we identified only varied through spacer deletions and yet they revealed 30 times more diversity than any of the other gene markers specifically selected for their polymorphism. Furthermore, unlike MLSA methods, which require multiple gene primers and may be biassed by paralogous sequences and homologous recombination if the gene markers are not carefully chosen, working with CRISPR requires a single set of primers, the orthology of the marker can be guaranteed through genomic context, and while homologous recombination within CRISPR has been observed, it appears to be extremely rare [83]. As an alternative to MLSA, whole genome shotgun sequencing is often preferred for assessing the genetic diversity of heterogeneous bacterial populations. Such an approach has the advantage of revealing the genetic diversity across whole genomes but, in addition to its higher cost (for endosymbionts the sequencing yield from metagenomes is largely reduced by host DNA contamination), this method cannot discriminate between strains at the level of individual bacteria. Thus, the smallest sampling unit is that of the metagenomic population; individual hosts in the case of symbiont studies. Hence, whole-genome shotgun sequencing would require extensive field sampling in order to resolve strain-level beta diversity. In contrast, with the CRISPR marker, one sequencing read represents one bacterial cell, which can be identified at the strain level. Therefore this method better harnesses the power of high-throughput sequencing for the purpose of strain-level population genetic studies particularly when dealing with unculturable bacterial species.

Nonetheless, there are a number of limitations to the use of CRISPR for DNA barcoding. First, not all prokaryote species possess the marker. The CRISPR-cas immunity is only present in about half of bacteria [64] and the system is rapidly lost in species undergoing reductive genome evolution such as vertically transmitted symbionts [63, 64]. Second, this marker is not appropriate for characterizing whole communities. Because of the great diversity of CRISPR-cas systems and CRISPR arrays (neither the flanker nor the repeat sequences are conserved across species), a universal primer may never be developed. Third, primer development for a single species necessitates a reference genome that includes the CRISPR genomic context. This is because whole CRISPR systems can be horizontally transferred across species [61, 84]. Hence, to ensure orthology of the amplified sequences, one of the primers has to target a region next to the array and outside of the operon. Finally, the cost of long-read high-throughput sequencing is still prohibitive. In this study, for the same effective depth of coverage, sequencing the CRISPR array cost roughly three times the amount needed for sequencing the smaller gene amplicons. However, third-generation sequencing technology costs are steadily falling and for well-characterized CRISPR arrays other, less onerous, genotyping methods exist (c.f. CRISPR-typing [17]). We therefore conclude that despite these limitations, CRISPR represents a promising tool for strain-tracking in a wide variety of uncultured bacteria.

## Supplementary Data

Supplementary material 2
